# Two Benzene Rings with a Boron Atom Comprise the Core Structure of 2-APB Responsible for the Anti-Oxidative and Protective Effect on the Ischemia/Reperfusion-Induced Rat Heart Injury

**DOI:** 10.3390/antiox10111667

**Published:** 2021-10-22

**Authors:** Yan-Cheng Shen, Yan-Jhih Shen, Wen-Sen Lee, Michael Yu-Chih Chen, Wei-Chia Tu, Kun-Ta Yang

**Affiliations:** 1 Ph.D. Program in Pharmacology and Toxicology, School of Medicine, Tzu Chi University, Hualien 97004, Taiwan; 103752101@gms.tcu.edu.tw (Y.-C.S.); 101327103@gms.tcu.edu.tw (Y.-J.S.); 2 Graduate Institute of Medical Sciences, College of Medicine, Taipei Medical University, Taipei 11031, Taiwan; wslee@tmu.edu.tw; 3 Department of Cardiology, Buddhist Tzu Chi General Hospital, Hualien 97002, Taiwan; chensmed@hotmail.com; 4 Master Program in Physiological and Anatomical Medicine, School of Medicine, Tzu Chi University, Hualien 97004, Taiwan; 103327101@gms.tcu.edu.tw; 5 Department of Physiology, School of Medicine, Tzu Chi University, Hualien 97004, Taiwan

**Keywords:** reactive oxygen species, myocardial infarction, ischemia/reperfusion, boron, antioxidant, cardioprotection

## Abstract

To identify the core structure of 2-aminoethoxydiphenyl borate (2-APB) responsible for the anti-oxidative and protective effect on the ischemia/reperfusion (I/R)-induced heart injury, various 2-APB analogues were analyzed, and several antioxidant assays were performed. Cell viability was determined using 3-(4,5-dimethylthiazol-2-yl)-2,5-diphenyltetrazolium bromide (MTT) assay. Myocardial infarct size was quantified using triphenyl tetrazolium chloride (TTC) staining. The levels of tumor necrosis factor-alpha (TNF-α) and cleaved-caspase-3 protein were evaluated as an indicator for the anti-inflammatory and anti-apoptotic effect, respectively. Our data show that 2-APB, diphenylborinic anhydride (DPBA) and 3-(diphenylphosphino)-1-propylamine (DP3A) all exerted the anti-oxidative activity, but only 2-APB and DPBA can scavenge H_2_O_2_. 2-APB and DPBA can potently inhibit hydrogen peroxide (H_2_O_2_)- and hypoxanthine/xanthine oxidase (HX/XOD)-induced increases in intracellular H_2_O_2_ and H9c2 cell death. 2-APB and DPBA were able to decrease the I/R-induced adult rat cardiomyocytes death, myocardial infarct size, and the levels of malondialdehyde (MDA) and creatine kinase-MB (CK-MB). Our results suggest that the two benzene rings with a boron atom comprise the core structure of 2-APB responsible for the anti-oxidative effect mediated through the reaction with H_2_O_2_ and generation of phenolic compounds, which in turn reduced the I/R-induced oxidative stress and injury in the rat heart.

## 1. Introduction

Acute myocardial infarction has been a significantly high cause of death in the world. Even if reperfusion is performed through primary percutaneous coronary intervention, the mortality and morbidity of patients remain high in death and heart failure at 1 year [1]. Previous studies have indicated that the ischemia/reperfusion (I/R) injury can be suppressed through ischemic preconditioning (IPC) and ischemic post-conditioning (IPost). Both IPC and IPost were shown to significantly protect the heart from the I/R injury [2,3,4]. The cardioprotective mechanisms induced by IPC and IPost can be potently activated by pharmacological conditioning to reduce the infarct size and improve clinical outcome of acute myocardial infarction after reperfusion [5]. In case of a burst of reactive oxygen species (ROS) produced in the first few minutes of myocardial reperfusion, antioxidants were shown to improve contractile function and/or reduce infarct size in myocardial I/R animal models [6]. Antioxidants can prevent biomolecules (proteins, nucleic acids, lipids, etc.) from undergoing oxidative damage caused by free radical-mediated reactions. They can inhibit oxidizing chain reactions in several ways, including direct scavenging of ROS, inhibition of enzyme activities, and chelating of metal ions (i.e., Fe^2+^ and Cu^2+^). Their beneficial effects are related to diseases, i.e., atherosclerosis, coronary heart disease, certain tumors, and aging, in which oxidative processes are remarkable [7,8].

Previous studies have found that 2-aminoethoxydiphenyl borate (2-APB) can effectively inhibit cardiac I/R injury. 2-APB, first synthesized by the esterification of diphenylboronic acid with aminoethanol in ethanol in 1958 [9], has been broadly used as a blocker of store-operated calcium channels, an inositol 1,4,5-trisphosphate receptor inhibitor, and a multiple transient receptor potential family of cation channels blocker [10,11], and has been shown to inhibit hydrogen peroxide (H_2_O_2_)-induced cell death by preventing the elevation of intracellular Ca^2+^ concentration in PC12 cells [12] and beta-cells [13]. In addition to the inhibition of cytosolic Ca^2+^ elevation, 2-APB was demonstrated to mitigate the I/R-induced organ damage (i.e., kidney [14], ovary [15], and testis [16]) through its anti-oxidative and anti-apoptotic effect in rats.

Although 2-APB was shown to inhibit cardiac I/R injury, it was reported to induce arrhythmia in Langendorff-perfused adult rat hearts [17,18]. Wang et al. have demonstrated that the 2-APB-induced rat heart arrhythmia links to the Orai protein that forms the pore of the store-operated calcium channel-related calcium entry [18]. Although 2-APB can inhibit inositol triphosphate receptors and TRPs, it was shown that a concentration of 2-APB that inhibit inositol triphosphate receptors or TRPs is not able to destabilize rat hearts [18]. In addition, 2-APB was demonstrated to possess potentiation and inhibition effects on store-operated calcium entry (SOCE) in BL41 cells, suggesting that the 2-APB-induced arrhythmia may be related to its effect on SOCE potentiation ability. Dellis et al. have used several 2-APB analogues to identify the main structure of 2-APB that plays an important role in SOCE potentiation ability [19]. Recently, Hirofumi et al. demonstrated that 2-APB can protect the heart against I/R-induced injury, at least partially by the scavenging of extracellular ROS in mice [20]. However, it is still unclear why 2-APB can be such an effective antioxidant and whether 2-APB has a special structure that can quickly interact with free radicals or directly scavenge free radicals.

It has been reported that 2-APB molecules contain three distinct parts: an ethanolamine chain, a boron-oxygen core, and two benzene rings [19]. Based on a previous study [19], we screened a small library of 2-APB analogues, which have different effects on SOCE. Diphenylborinic anhydride (DPBA), a borinate ester, like 2-APB, possesses SOCE potentiation and inhibition ability. Other 2-APB analogues, dimesitylborinic acid (DMBA), 3-(diphenylphosphino)-1-propylamine (DP3A), and diphenhydramine (DPH) only possess SOCE inhibition ability, but not potentiation. Phenylboronic acid (PBA) is not able to inhibit and potentiate SOCE [19]. The aim of this study was to use various 2-APB analogues to identify the core structure of 2-APB responsible for the anti-oxidative effect and the protective effect on the I/R-induced heart injury. Once we identified the core structure of 2-APB, 2-APB analogues, which possess ROS scavenging ability and protective effect on the I/R-induced heart injury but are not able to potentiate SOCE, could possibly be designed.

## 2. Materials and Methods

### 2.1. Chemicals

Chemicals and general laboratory reagents including H_2_O_2_, 2-APB, diphenylborinic anhydride (DPBA), phenylboronic acid (PBA), phosphate-buffered saline (PBS), 3-(diphenylphosphino)-1-propylamine (DP3A), dimesitylborinic acid (DMBA), diphenhydramine (DPH), SKF-96365, phenol, L-Ascorbic acid, hypoxanthine (HX), and xanthine oxidase (XOD) were purchased from Sigma-Aldrich (Saint Louis, MO, USA).

### 2.2. Cell Culture

Rat embryonic cardiomyoblast-derived H9c2 cells (American Type Culture Collection, Rockville, MD, USA) at a density of 6000 cells/cm^2^ were grown in 24-well plates in Dulbecco’s modified Eagle’s medium with high glucose (Thermo Fisher Scientific Inc., Cleveland, OH, USA) supplemented with 10% fetal bovine serum (FBS) and 1% penicillin/streptomycin in 95% air/5% CO_2_ at 37 °C. 

### 2.3. Cell Viability Measurement

Cell viability was determined using 3-(4,5-dimethylthiazol-2-yl)-2,5-diphenyltetrazolium bromide (MTT) assay. Briefly, cells were cultured in a 24-well plate. After treatment with indicated chemicals for 4 h, 500 μL of MTT (5 mg/mL in PBS) were then added in each well at 37 °C for 2 h, followed by 500 μL of dimethyl sulfoxide (DMSO) in each well to dissolve the formazan. The absorbance at 570 nm was measured by a spectrophotometer (Multiskan EX; Thermo Scientific, Rockford, IL, USA).

### 2.4. 2,2′-Azino-bis(3-ethylbenzothiazoline-6-sulfonic acid) (ABTS) Assay

To evaluate total antioxidant capacity of 2-APB analogues, 2,2′-azino-bis(3-ethylbenzothiazoline-6-sulfonic acid) (ABTS) assay was conducted following a standard method provided by Sigma (EC 1.11.1.7) with minor modifications. ABTS assay is a common method used to detect total antioxidant capacity. While ABTS reacts with H_2_O_2_ catalyzed by peroxidase, ABTS^●+^ radical (an oxidized ABTS) is generated and a green color appears. Briefly, each well of the 96-well plate received 10 μL of KH_2_PO_4_ (100 mM, pH 5.0), 6 μL of H_2_O_2_ (1.66 mM), and various concentrations of 4 μL of 2-APB (or its analogues), mixed, incubated for 5 min at room temperature (RT), added with 40 μL of 9.1 mM ABTS diammonium salt (Sigma-Aldrich, Saint Louis, MO, USA) and 40 μL of 1 unit/mL peroxidase (Sigma-Aldrich, Saint Louis, MO, USA), and then incubated for additional 5 min at RT. The green color that formed through the reaction was observed, and the absorbance values were measured at 405 nm using a spectrophotometer (Multiskan EX; Thermo Scientific, Rockford, IL, USA). An ascorbic acid-treated group was used as a positive control. The % inhibition was calculated by the formula (A_0_ – A_1_)/A_0_ × 100%, where A_0_ is the absorbance of the control and A_1_ is the absorbance in the presence of a test or standard sample.

### 2.5. Nitro-Blue Tetrazolium (NBT) Assay

The superoxide anion scavenging activity of 2-APB and its analogues was determined using the nitro-blue tetrazolium (NBT; Sigma-Aldrich, Saint Louis, MO, USA) reduction method as previously described with some modifications [21]. The superoxide anion was produced by KO_2_. In this experiment, a final volume of 25 μL of KH_2_PO_4_ (200 mM, pH 7.8), 10 μL of NBT (1 mM), 10 μL of indicated compounds, and 10 μL of saturated KO_2_ (dissolved in DMSO) were added in each well of the 96-well plate. The reaction mixture was placed at RT for 10 min, and the absorbance at 560 nm was determined against blank samples. Quercetin was used as a positive control for the NBT assay. The percentage of superoxide anion scavenging effect was calculated by the formula (A_0_ – A_1_)/A_0_ × 100%, where A_0_ is the absorbance of control reaction and A_1_ is the absorbance in presence of test or standard sample.

### 2.6. 1,1-Diphenyl-2-picryl hydrazyl (DPPH) Assay

Free radical scavenging activity of 2-APB and its analogues was measured using 1,1-diphenyl-2-picryl hydrazyl (DPPH; Sigma-Aldrich, St. Louis, MO, USA) as described previously [22] with small modifications. Briefly, 160 μL of DPPH (41.25 μM) in ethanol was mixed with 40 μL of various concentrations of indicated compounds in ethanol. The mixture was shaken vigorously, and then allowed to stand at RT for 30 min. The absorbance was measured at 517 nm using spectrophotometer (Multiskan EX; Thermo Scientific, Rockford, IL, USA). Ascorbic acid was used as a positive control for the DPPH assay. The percentage of DPPH scavenging effect was calculated by the formula (A_0_ – A_1_)/A_0_ × 100%, where A_0_ is the absorbance of control reaction and A_1_ is the absorbance in presence of test or standard sample.

### 2.7. H_2_O_2_ Scavenging Activity Assay

H_2_O_2_ scavenging activity of 2-APB and its analogues was measured using ferrous oxidation-xylenol orange (FOX) assay as previously described with minor modifications [23]. The FOX reagent was prepared by mixing 4.4 mM 2,6-di-tert-butyl-4-methylphenol (BHT) (HPLC grade methanol) with 1 mM xylenol orange (Sigma-Aldrich, Saint Louis, MO, USA) and 2.56 mM ammonium ferrous sulfate (dissolved in 0.25 M H_2_SO_4_; Sigma-Aldrich, Saint Louis, MO, USA) (9:1 *v*/*v*). In this experiment, 9 μL of indicated compounds (50 mM of stock concentration) and 9 μL of H_2_O_2_ (50 mM of stock concentration) were added in each well of the 96-well plate. The mixture was shaken, kept at RT for 30 min, and then 2 μL of methanol and 180 μL of FOX reagent were added and it was kept at RT for 30 min. The absorbance at 560 nm was measured using spectrophotometer. The % inhibition was calculated by the formula (A_0_ – A_1_)/A_0_ × 100%, where A_0_ is the absorbance of the control and A_1_ is the absorbance in the presence of the indicated compound.

### 2.8. Determination of Phenolic Content

The phenolic content of the generated compounds was determined using the Folin–Ciocalteu method as previously described with small modification [24,25]. Briefly, 10 mM of 2-APB, DPBA, or DP3A was mixed with 10 mM H_2_O_2_, and subsequently kept at RT for 10 min. Each group of the tested sample (10 μL) was then mixed with 75 μL of 10-fold diluted Folin–Ciocalteu reagent (Sigma-Aldrich, Saint Louis, MO, USA), and incubated at RT for 5 min. Then, 75 μL of 6% sodium carbonate was added, and the reaction mixture was kept at RT for 10 min before phenolic content was measured using a spectrophotometer at 760 nm. Gallic acid (Sigma-Aldrich, Saint Louis, MO, USA) dissolved in ethanol/water (75:25, *v*/*v*, 0.3% HCl) was used as the standard to evaluate total phenolic compounds.

### 2.9. Flow Cytometric Analysis of the Intracellular ROS

The H_2_O_2_-induced intracellular ROS increase was recorded by 2′, 7′-dichlorodihydrofluorescein diacetate, acetyl ester (CM-H_2_DCFDA, Molecular Probes, Eugene, OR, USA). H9c2 cells were trypsinized, and then incubated with CM-H_2_DCFDA for 30 min at 37 °C followed by a wash using PBS. The cells were then treated with either H_2_O_2_ (100 μM) or hypoxanthine/xanthine oxidase (HX/XOD) (HX: 0.2 mM, XOD: 2 mU/mL) (with or without indicated compounds) for 2 h. The intracellular levels of ROS were measured using a Gallios Flow Cytometer (Beckman Coulter, Indianapolis, IN, USA) with excitation/emission wavelengths of 488/540 nm.

### 2.10. Adult Rat Cardiomyocytes Isolation

Adult male Sprague Dawley rats (8–9 weeks old, 250–350 g) cardiomyocytes were isolated according to the protocol described by Lien et al. [26]. After the animals were anesthetized, the hearts were excised and mounted in a Langendorff perfusion apparatus; perfused with a warmed (37 °C) solution containing 10 mM 4-(2-hydroxyethyl)-1-piperazineethanesulfonic acid (HEPES), 137 mM NaCl, 4 mM KCl, 1.2 mM MgSO_4_, 1.2 mM KH_2_PO_4_, 1.25 mM CaCl_2_, and 10 mM glucose, which were gassed with 100% O_2_ to wash out blood (flow rate of 8 mL/min); and then perfused with digestion solution composed of 0.08% protease XIV, 0.2% collagenase type II, and 0.1% bovine serum albumin (BSA) in a Ca^2+^-free solution for 15 min. The cells were filtered and the calcium concentration was gradually restored to 1.25 mM. The isolated cells (approx. 13,000 cells) were seeded on sterilized laminin-coated coverslips or dishes for 1 h. Finally, culture medium was switched to M199 medium containing 2 g/L BSA, 0.62 g/L taurine, 0.39 g/L carnitine, 10 units/mL penicillin, 10 mg/L streptomycin, 0.65 g/L creatine, and 2.2 g/L NaHCO_3_, and the cells were incubated in a CO_2_ incubator (95% CO_2_ and 5% O_2_ at 37 °C).

### 2.11. Ischemia and Reperfusion Injury

#### 2.11.1. Cultured Cardiomyocytes Model

Simulated I/R in cultured cardiomyocytes was performed using a modified protocol according to the protocol described by Vila-Petroff et al. [27]. Briefly, cells were stabilized in Normal Tyrode (NT) buffer (10 mM HEPES, 130 mM NaCl, 1.2 mM MgCl_2_, 4.5 mM KCl, 2 mM CaCl_2_, and 11 mM glucose) for 10 min. Ischemia was produced by incubating with an ischemia mimetic solution (20 mM HEPES, 123 mM NaCl, 0.5 mM MgSO_4_, 8 mM KCl, 2.5 mM CaCl_2_, 0.9 mM NaH_2_PO_4_, and 20 mM Na-lactate, gassed with 100% N_2_ and pH adjust to 6.8) for 30 min followed by reperfusion for 2 h with culture medium, and the cells were incubated in a CO_2_ incubator in an atmosphere of 95% CO_2_ and 5% O_2_ at 37 °C.

#### 2.11.2. Langendorff-Perfused Rat Hearts

Simulated I/R in rat hearts was performed using a modified protocol described by Lin et al. [28]. Rats were anesthetized with urethane (1.5 g/kg, i.p.) and the hearts were isolated and perfused in a Langendorff apparatus with a Krebs–Henseleit bicarbonate buffer (118 mM NaCl, 25 mM NaHCO_3_, 4.7 mM KCl, 1.2 mM MgSO_4_, 1.2 mM KH_2_PO_4_, 1.25 mM CaCl_2_, 11 mM Glucose, and pH = 7.4) at 37 °C. Krebs solution was gassed with 95% O_2_ and 5% CO_2_. After 20 min of stabilization, the hearts of the rats were subjected to 30 min of global ischemia, followed by 60 min of reperfusion with or without 2-APB (5 μM), DPBA (5 μM), or DP3A (5 μM) in Krebs–Henseleit bicarbonate buffer. The treatment concentrations of 2-APB, DPBA and DP3A were based on a previous study [17]. The hearts were frozen at −20 °C for 20 min, and then cut into slices (2 mm thick) along the long axis of the left ventricular (LV) from apex to base. Triphenyl tetrazolium chloride (TTC) staining was used to evaluate myocardial infarct size. The heart slices were incubated in 1% TTC PBS solution, pH 7.4, at 37 °C for 15 min. Tissues were then fixed in 4% PBS-buffered formalin overnight at RT, followed by photographing with the digital camera. The percentage of infarct size was calculated by ImageJ software (National Institutes of Health, Bethesda, MD, USA).

#### 2.11.3. In Vivo Rat I/R Model

Sixteen male Sprague–Dawley rats (400–500 g) were randomly divided into 4 groups: (1) sham group, (2) I/R group, (3) I/R + 2-APB group, and (4) I/R + DPBA group. Four animals were in each group. The animals were anesthetized, and the core temperature was maintained at 37.5 °C during surgery. The I/R injury model was established through the ligation of the left anterior descending (LAD) branch. The coronary artery was occluded for 30 min, followed by 2 h of reperfusion. After thoracotomy, a 6–0 silk suture was tied around LAD, and a small piece of PE50-polyethylene tubing was used to secure the ligature without damaging the artery to induce ischemia for 30 min, followed by 2 h reperfusion. Fifteen minutes before the PE50 tube was removed, the I/R + 2-APB group and the I/R + DPBA group were given 3 mg/kg of 2-APB (i.p.) and DPBA (i.p.), respectively. The 2-APB and DPBA treatment regimen was as previously described [15]. After reperfusion for 2 h, LAD was re-ligated, and 2 mL of 3% Evans blue dye was injected via the vein. After 5 min of staining, the heart was isolated, frozen at −20 °C for 20 min, cut into slices (2 mm thick), incubated in 1% TTC solution at 37 °C for 15 min, and then fixed in 4% formaldehyde. The slices were scanned, and the risk area (red) and the infarct size (white) were determined using ImageJ software (Version 1.48, National Institutes of Health, USA).

### 2.12. Lipid Peroxidation Determination

The lipid peroxidation was determined using MDA measurement [29]. Aliquots (25 mg from each group) from the heart tissue homogenate were mixed with 2,6-Di-tert-butyl-4-methylphenol-trichloroacetic acid (BHT-TCA) solution containing 1% BHT (*w*/*v*; Sigma-Aldrich, Saint Louis, MO, USA) dissolved in 20% TCA (*w*/*v*; Sigma-Aldrich, Saint Louis, MO, USA), and centrifuged (1000× *g* for 10 min at 4 °C), and then 0.5 mL of the supernatant was mixed with 1 mL solution containing 0.5 N HCl and 120 mM TBA buffered in 26 mM Tris. The mixture was heated at 80 °C for 10 min. After cooling, the absorbance of the resultant supernatant was read at 532 nm. MDA standard was prepared by dissolving 1,1,3,3 tetraethoxypropane (TEP; Sigma-Aldrich, Saint Louis, MO, USA) in water to give stock solution. The working solution was prepared by hydrolysis of 1 mL TEP stock solution in 50 mL 1% sulfuric acid and incubation for 2 h at RT. The resulting MDA standard was further diluted with 1% sulfuric acid to yield the final concentration to obtain the standard curve for the estimation of total MDA [30].

### 2.13. Western Blot Analysis

The isolated rat hearts were frozen at −80 °C. The frozen tissues were lysed with Radioimmunoprecipitation assay (RIPA) buffer (Merck Millipore, Burlington, MA, USA) containing 1% protease inhibitors (Calbiochem, San Diego, CA, USA) and centrifuged (10,600× *g* for 10 min at 4 °C). After the tissues were homogenized, the determination of protein concentration was performed by the Bradford assay with BSA as a standard. The samples were heated, and then loaded equally for separation using sodium dodecyl sulfate-polyacrylamide gel electrophoresis (SDS-PAGE). Proteins were electrophoretically transferred to polyvinylidene fluoride (PVDF) membranes. Five percent BSA solution was used to block the non-specific binding. Protein samples were probed with tumor necrosis factor-alpha (TNF-α) (1:2000; Cat. #3707, Cell Signaling Technology, Danvers, MA, USA), or cleaved-caspase-3 (1:1000; Cat. #C8487, Sigma-Aldrich, Saint Louis, MO, USA) overnight at 4 °C followed by their corresponding secondary antibodies for 1 h. Bands were visualized via an enhanced chemiluminescence kit (PerkinElmer, Waltham, MA, USA). The blots were developed using UVP Biospectrum 810 (Analytik Jena US LLC, Upland, CA, USA) and quantified by ImageJ software (Version 1.48, National Institutes of Health, USA).

### 2.14. Myocardial Injury Enzyme Evaluation

Following the reperfusion for 2 h, the blood sample was collected and centrifuged at 3000× *g* (4 °C, 15 min). The contents of creatine kinase-MB (CK-MB) in the supernatant were evaluated using the rat ELISA kit (MyBioSource, San Diego, CA, USA) following the manufacturer’s instructions.

### 2.15. Statistical Analysis

Data are expressed as mean ± standard errors (SEM). The statistical significance was evaluated by one-way ANOVA with Holm–Sidak mean comparison using a statistical program (Sigma Plot version 11.0 for Windows, Systat Software Inc, Chicago, IL, USA). A *p* value < 0.05 was considered statistically significant.

## 3. Results

### 3.1. Involvement of 2-APB-Sensitive Channels in the Protective Effect of 2-APB on the H_2_O_2_-Induced Cell Death

**Figure 1:**
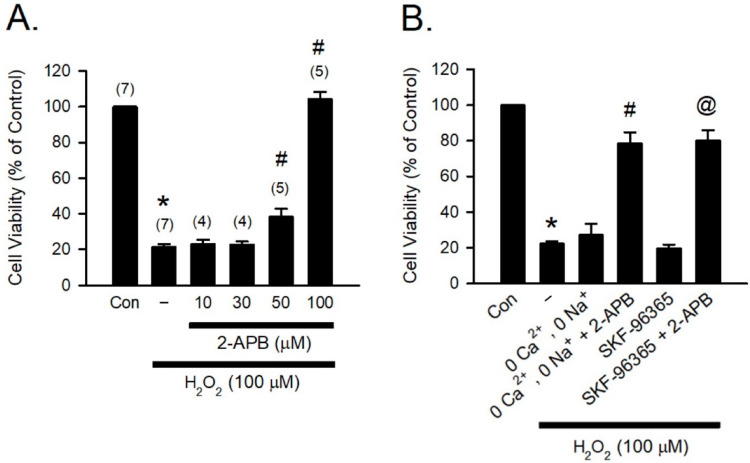
2-APB protects the H_2_O_2_-induced H9c2 cell death. (**A**) 2-APB at concentrations of 50 and 100 μM significantly attenuated the H_2_O_2_-induced cell death. Values shown in parenthesis represent the number in each group. * *p* < 0.05 different from control; ^#^ *p* < 0.05 different from H_2_O_2_. (**B**) Treatment with a transient receptor potential canonical (TRPC) channel blocker-SKF-96365 (10 μM) or incubation in the Ca^2+^ and Na^+^ free medium could not protect the H_2_O_2_-induced cell death. However, treatment 4 h with 2-APB and SKF-96365 together or 2-APB in the Ca^2+^ and Na^+^ free medium significantly reduced the H_2_O_2_-induced cell death. (*n* = 5). * *p* < 0.05 different from control; ^#^ *p* < 0.05 different from the 0 Ca^2+^, 0 Na^+^ + H_2_O_2_ group; ^@^ *p* < 0.05 different from the SKF-96365 + H_2_O_2_ group. Data are expressed as mean ± SEM. Abbreviations: Con, control; SKF-96365, 1{β-[3-(4-methoxyphenyl)propoxyl]-4-methoxyphenethyl}-1H-imidazole hydrochloride; 2-APB, 2-aminoethoxydiphenyl borate; −, only H_2_O_2_ (100 μM).

H_2_O_2_, one of the most abundant and stable forms of ROS, has been implicated in causing inflammation, cellular dysfunction, and apoptosis, leading to tissue and organ damage [31]. Previous studies have reported that 2-APB can potently protect various types of cells against H_2_O_2_-induced cell death [12,32,33]. To study the cytoprotective effect of 2-APB, H9c2 cells were co-treated with 2-APB and H_2_O_2_ (100 μM) for 4 h, and the cell viability was assessed using MTT assay. Following previous studies by Hirofumi et al. [20], the concentrations of 2-APB between 10–100 µM were used in our initial studies. Since 2-APB at a concentration of 50 and 100 µM was shown to possess significant protective effect (Figure 1A), 100 µM of 2-APB was used for subsequent experiments. It has been shown that increases in intracellular Ca^2+^ and Na^+^ concentration and potentiation of multiple transient receptor potential canonical (TRPC) channels are associated with the H_2_O_2_-induced cell death, and 2-APB can inhibit TRPC channels [20,34,35]. However, in the present study, removal of the extracellular Ca^2+^ and Na^+^ or treatment with a TRPC channel blocker-SKF-96365 failed to prevent the H_2_O_2_-induced cell death (Figure 1B). Treatment with 2-APB and SKF-96365 together or 2-APB in the Ca^2+^ and Na^+^ free medium significantly reduced the H_2_O_2_-induced cell death, suggesting that the influx of extracellular Ca^2+^ and Na^+^ and TRPC channel are not involved in the 2-APB-mediated protective effect against the H_2_O_2_-induced cell death. 

### 3.2. Antioxidant Activity of 2-APB Analogues

**Figure 2:**
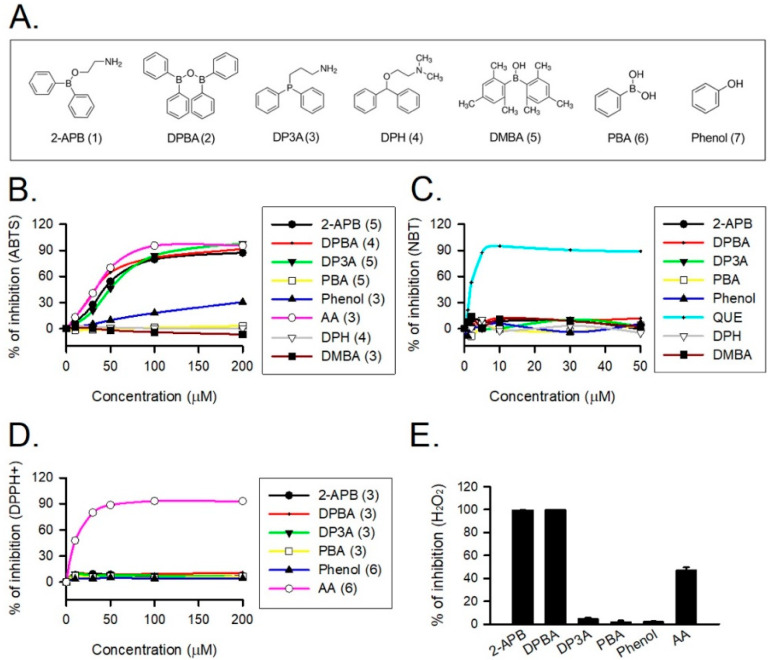
Reactive oxygen species (ROS) scavenging ability of 2-APB and its analogues. (**A**) Structure of 2-APB (compound 1), 2-APB analogues (compound 2–6), and phenol used in this study. (**B**) 2-APB, diphenylborinic anhydride (DPBA), 3-(diphenylphosphino)-1-propylamine (DP3A), and phenol were able to inhibit 2, 2′-azino-bis(3-ethylbenzothiazoline-6-sulfonic acid) (ABTS) radical. Values shown in parenthesis represent the number in each group. (**C**) 2-APB, DPBA, and DP3A were not able to inhibit superoxide anion. (*n* = 3). (**D**) 2-APB, DPBA, and DP3A were not able to inhibit DPPH radical. Values shown in parenthesis represent the number in each group. (**E**) 2-APB and DPBA, but not DP3A, were able to inhibit H_2_O_2_. (*n* = 3). Abbreviations: AA, ascorbic acid; QUE, quercetin. DPH, diphenhydramine; PBA, phenylboronic acid; DMBA, dimesitylborinic acid.

2-APB was demonstrated to protect cells against the H_2_O_2_-induced death through direct scavenging of ROS [20]. To verify the correlation between anti-oxidative effect and structure of 2-APB analogues, the total antioxidant capacity of the tested compounds (Figure 2A) was evaluated using ABTS assay. NBT, DPPH, and FOX assays were conducted to assess superoxide anion, DPPH radical, and H_2_O_2_ scavenging abilities of 2-APB analogues, respectively. Our data revealed that 2-APB, DPBA, and DP3A could significantly scavenge ABTS radicals (Figure 2B), indicating the anti-oxidative activity of 2-APB, DPBA, and DP3A. All compounds failed to scavenge superoxide anion (Figure 2C) and DPPH radicals (Figure 2D). Only 2-APB and DPBA were able to scavenge H_2_O_2_ (Figure 2E). Collectively, these results suggest that the ROS scavenging ability of 2-APB and DPBA may be relatively specific to H_2_O_2_.

### 3.3. Generation of Phenolic Compounds by 2-APB Analogues

**Figure 3:**
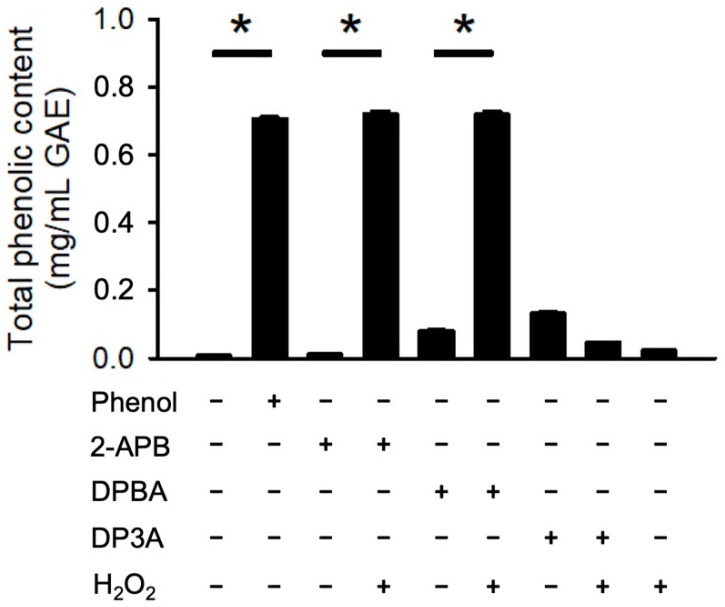
Generation of phenolic compounds by 2-APB analogues. 2-APB and DPBA could react with H_2_O_2_ and generate phenolic compound_._ Generation of phenolic compounds were detected by Folin–Ciocalteu reagent (FCR). (*n* = 3). * *p* < 0.05. Data are expressed as mean ± SEM.

It has been demonstrated that borate esters can interact with peroxides, hence generating phenolic antioxidant [36,37,38]. To evaluate whether 2-APB, DPBA, and DP3A could react with H_2_O_2_ and generate phenolic compounds, a Folin–Ciocalteu reagent assay was conducted. Our data showed that only 2-APB and DPBA could react with H_2_O_2_ and generate phenolic compounds (Figure 3). These findings are consistent with previous studies showing that 2-APB and DPBA, which are aryl-borates (boric acids with aromatic ring), could interact with H_2_O_2_ and generate phenolic compounds [36,39].

### 3.4. Scavenging Activities of 2-APB Analogues on Intracellular ROS

**Figure 4:**
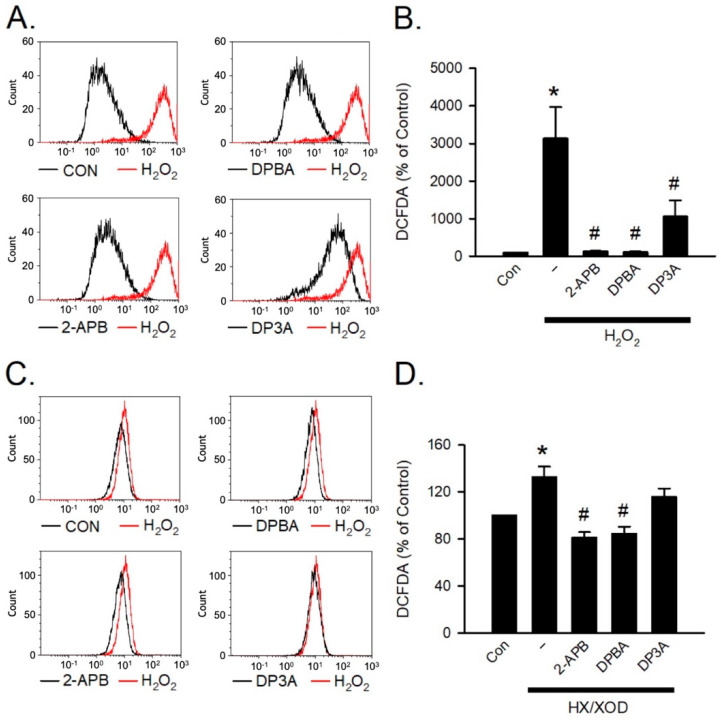
Scavenging effect of 2-APB analogues on intracellular H_2_O_2_ elevation. Flow cytometric analyses show scavenging effect of 2-APB analogues on intracellular H_2_O_2_ elevation determined by activity of the fluorescent probe, 2′, 7′-dichlorodihydrofluorescein diacetate, acetyl ester (CM-H_2_DCFDA) following simultaneous incubation with 2-APB analogues and H_2_O_2_ or hypoxanthine/xanthine oxidase (HX/XOD) for 2 h. (**A**) Representative flow cytometry figures show the scavenging effects of 2-APB analogues on the intracellular ROS elevation in the cells exposed to H_2_O_2_. (**B**) 2-APB, DPBA, and DP3A at a concentration of 100 μM inhibited the intracellular H_2_O_2_ elevation in the cells exposed to H_2_O_2_ (100 μM). (*n* = 7). * *p* < 0.05 different from control; ^#^ *p* < 0.05 different from the H_2_O_2_ group. (**C**) Representative flow cytometry histograms show the scavenging effects of 2-APB analogues on the intracellular H_2_O_2_ elevation induced by HX/XOD (HX: 0.2 mM, XOD: 2 mU/mL). (**D**) 2-APB and DPBA, but not DP3A, inhibited the HX/XOD-induced intracellular H_2_O_2_ elevation. (*n* = 7). * *p* < 0.05 different from control; ^#^ *p* < 0.05 different from the HX/XOD group. Data are expressed as mean ± SEM. Abbreviations: Con, control.

H_2_O_2_ and hypoxanthine/xanthine oxidase (HX/XOD) are commonly used for inducing oxidative stress [40]. To investigate the inhibition effect of 2-APB analogues on the intracellular H_2_O_2_, the HX/XOD system was used. Briefly, a superoxide anion was generated by HX/XOD system, and subsequently transformed into H_2_O_2_ by superoxide dismutase (SOD). To assess whether 2-APB, DPBA, and DP3A could scavenge intracellular H_2_O_2_, flow cytometric analysis was conducted using H_2_DCFDA (a H_2_O_2_ indicator). Our data showed that 2-APB, DPBA, and DP3A could inhibit the intracellular H_2_O_2_ elevation in the cells exposed to H_2_O_2_ (100 μM) (Figure 4A,B), whereas the HX/XOD-induced intracellular H_2_O_2_ elevation could only be inhibited by 2-APB and DPBA, but not by DP3A (Figure 4C,D).

### 3.5. Protective Effects of 2-APB, DPBA, and DP3A on the H_2_O_2_- and HX/XOD-Induced H9c2 Cell Death

**Figure 5:**
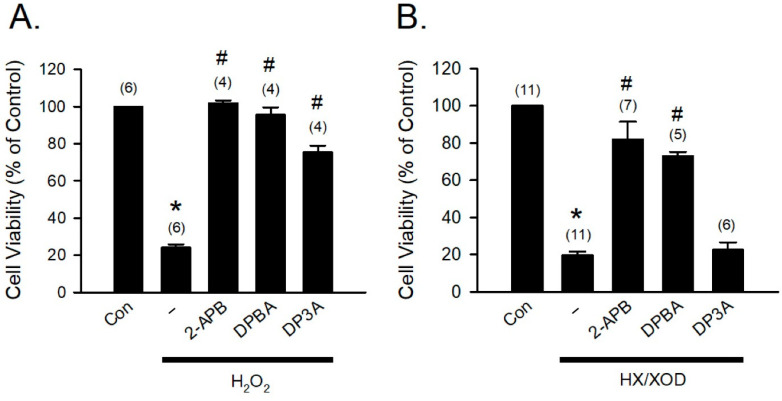
Protective effects of 2-APB, DPBA, and DP3A on the H_2_O_2_- or HX/XOD-induced cell death in H9c2 cells. (**A**) 2-APB, DPBA, and DP3A at a concentration of 100 μM significantly inhibited the H_2_O_2_ (100 μM)-induced cell death. * *p* < 0.05 different from control; ^#^ *p* < 0.05 different from the H_2_O_2_ group. (**B**) 2-APB (100 μM) and DPBA (100 μM), but not DP3A (100 μM), significantly inhibited the cell death induced by HX/XOD (HX: 0.2 mM, XOD: 2 mU/mL). * *p* < 0.05 different from control; ^#^ *p* < 0.05 different from the HX/XOD group. Values shown in parenthesis represent the number in each group. Data are expressed as mean ± SEM. Abbreviations: Con, Control; HX/XOD, hypoxanthine/xanthine oxidase.

To further confirm that the H_2_O_2_- and HX/XOD-induced cell death can be prevented by 2-APB, DPBA, and DP3A, we applied H_2_O_2_ and HX/XOD to induce H9c2 cell death, and then cell viability was determined using MTT assay. Our data revealed that 2-APB, DPBA, and DP3A significantly inhibited the H_2_O_2_-induced cell death (Figure 5A), while the HX/XOD-induced cell death could only be inhibited by 2-APB and DPBA, but not by DP3A (Figure 5B).

### 3.6. Cardioprotective Effects of 2-APB and Its Analogues

**Figure 6:**
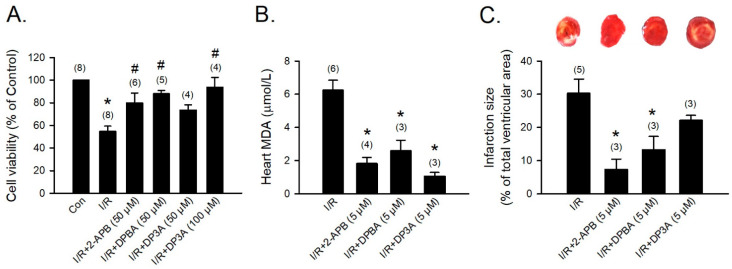
Protective effects of 2-APB, DPBA, and DP3A on the I/R-induced cell death, myocardial infarction, and lipid peroxidation. (**A**) 2-APB, DPBA, and DP3A inhibited the I/R-induced cell death. Isolated adult rat cardiomyocytes were exposed to ischemia for 30 min, followed by 2 h reperfusion with 2-APB, DPBA, or DP3A. MTT assay was used to determine cell viability. * *p* < 0.05 different from control; ^#^ *p* < 0.05 different from the I/R group. (**B**) 2-APB, DPBA, and DP3A inhibited lipid peroxidation after I/R. * *p* < 0.05 different from the I/R group. (**C**) 2-APB and DPBA, but not DP3A, decreased myocardial infarct size induced by I/R. Isolated rat hearts were perfused by a Langendorff system. The hearts were stable for 20 min, and then exposed to 30 min ischemia followed by 1 h reperfusion with 2-APB, DPBA, or DP3A. Myocardial infarct size was determined by Triphenyl tetrazolium chloride (TTC) staining. The infarct size was expressed as a percentage of left ventricular volume for each heart. * *p* < 0.05 different from the I/R group. Values shown in parenthesis represent the number in each group. Data are expressed as mean ± SEM. Abbreviations: I/R, ischemia/reperfusion; MDA, malondialdehyde.

To evaluate the protective effect of 2-APB analogues on the I/R-induced heart injury, 2-APB, DPBA, and DP3A were administrated during the period of I/R of the adult rat cardiomyocyte model. Treatment with 2-APB (50 μM), DPBA (50 μM), or DP3A (100 μM) protected adult rat cardiomyocytes against the I/R-induced cell death (Figure 6A). Myocardial infarct size and lipid peroxidation were evaluated using TTC staining and MDA measurement, respectively, to verify the protective effect of 2-APB analogues on the I/R-induced heart damage. Treatment with 2-APB, DPBA, and DP3A significantly inhibited lipid peroxidation (Figure 6B), but only 2-APB and DPBA significantly decreased myocardial infarct size (Figure 6C).

**Figure 7:**
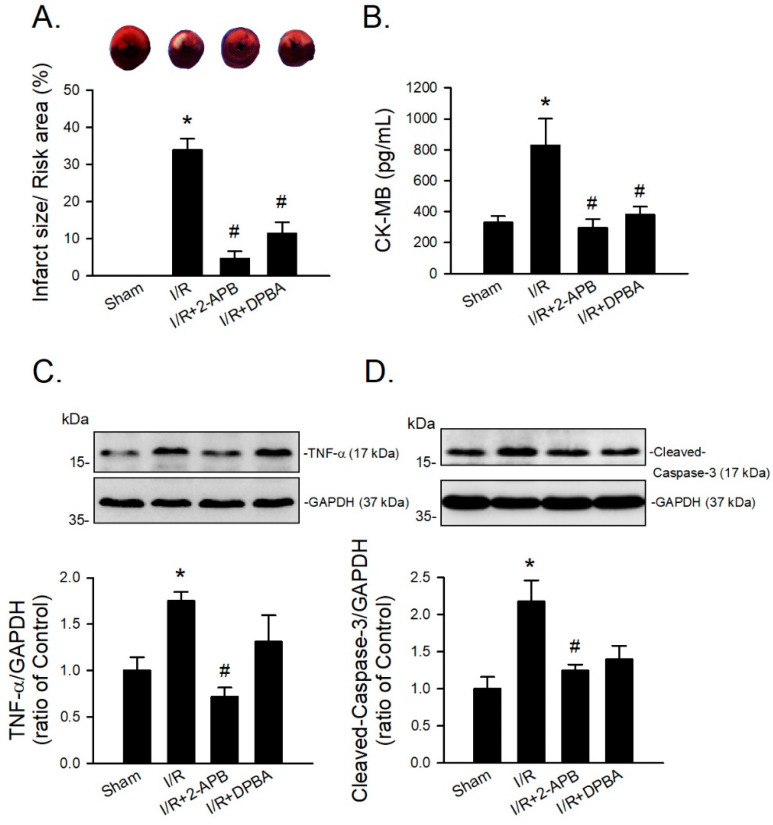
Protective effects of 2-APB and DPBA on the I/R-induced myocardial infarction, inflammation, and apoptosis in an in vivo I/R model. (**A**) Both 2-APB and DPBA at a concentration of 3 mg/kg significantly decreased infarct size/risk area induced by I/R. (*n* = 4). (**B**) Both 2-APB and DPBA at a concentration of 3 mg/kg significantly decreased the levels of creatine kinase-MB (CK-MB). (*n* = 4). 2-APB (3 mg/kg) significantly reduced the levels of TNF-α (**C**), and cleaved-caspase-3 (**D**). Top panel shows representative Western blot analyses. Bottom panel shows graphs of quantification of these data adjusted with GAPDH protein level and expressed as ratio over the corresponding control. (*n* = 4). * *p* < 0.05 different from the sham group; ^#^ *p* < 0.05 different from the I/R group. Data are expressed as mean ± SEM. Abbreviations: CK-MB, creatine kinase-MB; GAPDH, glyceraldehyde 3-phosphate dehydrogenase; I/R, ischemia/reperfusion; TNF-α, tumor necrosis factor-α.

Since DP3A showed a less protective effect on the I/R-induced myocardial infarction, 2-APB and DPBA were selected to further evaluate their protective effect on the I/R-induced injury in an in vivo model. CK-MB is a reliable marker of myocardial injury. Our data revealed that both 2-APB and DPBA effectively reduced the ratio of infarct size/risk area (Figure 7A) and the levels of CK-MB in the I/R-injured heart (Figure 7B). To investigate the anti-inflammatory and anti-apoptotic effect of 2-APB and DPBA on the I/R-induced cardiac damage, the protein levels of TNF-α and cleaved-caspase-3 were detected using Western blot analysis. Our data showed that only 2-APB significantly reduced the levels of TNF-α and cleaved-caspase-3 (Figure 7C,D).

## 4. Discussion

In the present study, we demonstrated that 2-APB and DPBA, but not DP3A, can scavenge H_2_O_2_ and decrease the heart infarct size through their anti-oxidative effect. Previous studies have revealed that the protective effect of 2-APB on the H_2_O_2_-induced death in PC12 cells [12] and beta cells [13] is mediated through inhibiting an increase in intracellular Ca^2+^ concentration. However, our data revealed that SKF-96365 (a TRPC channel blocker) was not able to inhibit the H_2_O_2_-induced cell death (Figure 1B). Whether the protective effect of 2-APB on the H_2_O_2_-induced cell death is related to the 2-APB sensitive channel or receptor remains controversial. Hirofumi et al. indicated that the protective effect of 2-APB on H_2_O_2_-induced cell death is not related to the TRPM3/7 and TRPC channel [20], while Masahiro et al. demonstrated that SKF-96365 protects INS-1 beta-cells against the H_2_O_2_-induced injury [34]. Our results suggested that the influx of extracellular Ca^2+^ and Na^+^ and TRPC channel were not involved in the 2-APB-mediated protective effect against the H_2_O_2_-induced cell death (Figure 1B). The ROS scavenging effect of 2-APB has been implicated in the protective effect against the H_2_O_2_-induced cell death [20]. These findings suggest that besides the inhibition of intracellular Ca^2+^ elevation, the ROS scavenging effect might be involved in the protective effect of 2-APB on the H_2_O_2_-induced cell death. 2-APB has been demonstrated to contain three distinct parts: an ethanolamine chain, a boron-oxygen core, and two benzene rings [19]. To identify the core structure of 2-APB responsible for the anti-oxidative effect, we screened a small library of 2-APB analogues based on a previous study on the core structure responsible for the effects of 2-APB on the SOCE potentiation [19]. Our results revealed that both 2-APB and DPBA can scavenge H_2_O_2_ (Figure 2E), while all of 2-APB analogues examined in this study were not able to inhibit superoxide anion and DPPH radicals (Figure 2C,D). Regarding the scavenging ability, 2-APB and DPBA might be relatively specific to H_2_O_2_ as compared with superoxide anion and DPPH radicals. Since DPBA does not possess ethanolamine chain but could scavenge ABTS radicals and H_2_O_2_, we speculate that ethanolamine chain might not be related to the anti-oxidative effect of 2-APB. Furthermore, DMBA, DPH, and PBA cannot inhibit ABTS radicals (Figure 2B), suggesting that a boron or phosphorus atom with two benzene rings might be the main structure of 2-APB analogues to possess anti-oxidative effect. It has been shown that 2-APB can directly scavenge extracellular ROS through chemical reaction with H_2_O_2_ and generation of phenol, thereby exhibiting an anti-oxidative effect [20]. In addition, aryl-borate esters were demonstrated to be easily hydrolyzed to generate corresponding phenols by reacting with H_2_O_2_ [36,37,38]. Our data revealed that 2-APB and DPBA, but not DP3A, generated phenols after reacting with H_2_O_2_ (Figure 3), suggesting that the anti-oxidative mechanism of DP3A might be different from 2-APB and DPBA. The reaction between borate esters and H_2_O_2_ have been applied in the development of nanoparticle for drug delivery and imaging cellular ROS. They were used to conjugate anti-cancer drugs to hide the active site until they reach the ROS-rich environment (i.e., tumor and inflamed tissue), where borate esters are degraded to expose the active site of drug molecules [31,37,41]. They have also been applied to detect the intracellular H_2_O_2_ concentrations in living cells [31,42,43]. In addition, it has been reported that aryl-borate esters are able to destroy hydroperoxides, and subsequently generate alcohol and the corresponding boric acid ester [39]. This evidence further suggests that 2-APB might be a secondary antioxidant, which is frequently referred to as hydroperoxide decomposers, acting to convert hydroperoxides into non-radical, non-reactive, and stable products [44].

Our data revealed that 2-APB and DPBA, but not DP3A, significantly inhibited the HX/XOD-induced intracellular H_2_O_2_ increases (Figure 4C,D). Moreover, 2-APB and DPBA also inhibited the H_2_O_2_- and HX/XOD-induced cell death. Collectively, although 2-APB and DPBA had no or little effect on scavenging of superoxide anion (Figure 2C), they could decrease the intracellular H_2_O_2_ increases (Figure 4C,D), thus protecting the HX/XOD-induced cell death (Figure 5B). Our data also demonstrated that 2-APB, DPBA, and DP3A could protect cells against the I/R-induced death (Figure 6A), and they significantly inhibited lipid peroxidation (Figure 6B). However, only 2-APB and DPBA, but not DP3A, could decrease the heart infarct size (Figure 6C). Comparing with 2-APB and DPBA, DP3A was shown to possess a lesser effect on the inhibition of HX/XOD-induced increases in intracellular H_2_O_2_ (Figure 4D) and cell death (Figure 5B), and decreases in heart infarct size (Figure 6C). It has been indicated that both phenylphosphonite (an organophosphorus compound with aromatic ring) and phenylboronate (a boric acid with aromatic ring) are efficient antioxidants. Moreover, phenylboronate possesses more anti-oxidative activity compared to phenylphosphonite [36]. Therefore, we suspect that the less protective effect of DP3A might be partly because DP3A contains phosphorus atom instead of boron atom. In the in vivo I/R model, both 2-APB and DPBA were shown to significantly reduce the infarct size/risk area (Figure 7A), and CK-MB (Figure 7B). However, our data revealed that 2-APB, but not DPBA, significantly reduced the protein levels of TNF-α (Figure 7C) and cleaved-caspase-3 (Figure 7D). Since the occurrence of acute myocardial infraction, resulting in a decrease in contractile function, has been known to be closely related to inflammation [45], the contractile function of the heart after I/R injury was further evaluated by echocardiography. Our data showed that 2-APB significantly increased the recovery of EF and FS in the rat heart after I/R injury (Figure S1).

Regarding the effect of 2-APB on Ca^2+^ homeostasis, Baldwin et al. used 2-APB as a scaffold to design a series of novel boron compound analogues, and found that the compounds, which inhibit inflammation, have no effect on Ca^2+^ homeostasis [46]. It has been shown that both DPBA and 2-APB have a dual effect (inhibition and potentiation) on SOCE in BL41 cells, and DPBA appears to potentiate the SOCE activity with a greater efficacy than 2-APB [19], suggesting that DPBA is also able to affect Ca^2+^ homeostasis. Although DPBA could not significantly inhibit the expression of TNF-α (Figure 7C) and cleaved-caspase-3 (Figure 7D), DPBA possesses the anti-oxidative activity and protective effect similar to 2-APB on the I/R-induced heart injury.

It has been reported that the 2-APB-induced arrhythmia is linked to Orai protein-related calcium entry. In addition, 2-APB has been indicated to potentiate SOCE, suggesting that 2-APB-induced arrhythmia is related to its effect on the SOCE potentiation ability. Unfortunately, our data showed that among the 2-APB analogues used, only 2-APB and DPBA, which have been indicated to potentiate SOCE, effectively scavenge ROS and protect against I/R-induced damage.

A previous study demonstrated that the main structure of 2-APB responsible for the SOCE potentiation is the boron oxygen core [19], while our present study showed that with two benzene rings with a boron atom make up the core structure of 2-APB responsible for the anti-oxidative and protective effect on the I/R-induced heart damage. These findings indicate that it is conflicting to scavenge ROS and protect the I/R-induced injury while not potentiating SOCE. However, in our study, in addition to 2-APB and DPBA, although DP3A was not able to reduce infarct size and scavenge H_2_O_2_, DP3A showed abilities to protect H9c2 cells and rat cardiomyocytes against the H_2_O_2_- and I/R-induced damage, respectively. Therefore, in addition to the boron atom with two benzene rings, a phosphorous atom with two benzene rings could also be used as a scaffold for the development of cardioprotective drugs without an effect on calcium homeostasis in the future.

There are some limitations in our study. Mitochondrial calcium uniporter (MCU) is one of the 2-APB sensitive channels [47], and Ru360 is a common MCU inhibitor. However, György et al. demonstrated that Ru360 fails to inhibit MCU in intact H9c2 cells [48]. A recent study reported a new MCU inhibitor-Ru265 that is cell-permeable and more potent than Ru360 [49]. However, since Ru265 is not commercially available and not easy to obtain, we could not test the involvement of MCU in the H_2_O_2_-induced H9c2 cell death. Whether MCU is involved in the protective effect of 2-APB on the H_2_O_2_-induced H9c2 cell death remains unsolved and deserves further investigation. Nevertheless, our study revealed that the anti-oxidative effect of 2-APB plays an important role in protecting the ROS-induced cell death. 2-APB at a concentration of 5 μM, which has been indicated to be not able to destabilized hearts [17], was used in our experiments. Since we did not detect heart beats during our experiment (in the Langendorff and in vivo model), we could not exclude the possibility that 2-APB could induce arrhythmia in rat hearts. This suggests that the results of the protective effects of 2-APB on I/R-induced heart damage might also be manifested when 2-APB induces arrhythmia. Therefore, using a boron or phosphorous atom with two benzene rings as scaffold to develop cardioprotective drugs that do not affect calcium homeostasis is worthy of further exploration in the future.

## 5. Conclusions

In conclusion, our data from the present study revealed that two benzene rings with a boron atom comprise the core structure of 2-APB responsible for the anti-oxidative effect through the reaction with H_2_O_2_ and generation of phenolic compounds, subsequently reducing the I/R-induced oxidative stress and injury in rat heart.

## Data Availability

Data is contained within the article and supplementary material.

## Supplementary Materials

The following are available online at https://www.mdpi.com/article/10.3390/antiox10111667/s1, Figure S1: In vivo rat I/R model for echocardiography.
