# Supplementary material for: Two Benzene Rings with a Boron Atom Comprise the Core Structure of 2-APB Responsible for the Anti-Oxidative and Protective Effect on the Ischemia/Reperfusion-Induced Rat Heart Injury
Source: Antioxidants (Basel). 2021 Oct 22;10(11):1667. doi: 10.3390/antiox10111667 (PMC8614801; doi:10.3390/antiox10111667)
Supplement: Supplementary file 1 [file antioxidants-10-01667-s001.zip › antioxidants-1386111-supplementary.pdf]

Supplementary data

Materials and Methods

In vivo rat I/R model for echocardiography

Briefly, 15 male Sprague-Dawley rats (400-500 g) were randomly divided into three groups: (1) sham group (n = 5), (2) I/R group (n = 5), and (3) I/R+2-APB group (n = 5). The animals were anesthetized, and the core temperature was maintained during surgery. After thoracotomy, LAD was tied by a 6-0 silk suture, and a small piece of PE50-polyethylene tubing was used to induce ischemia for 30 min. Fifteen min before PE50 tube was removed, the I/R+2-APB group was given 3 mg/kg 2-APB (i.p.). The 2-APB treatment regimen was as previously described [15]. After 30 min, the 6-0 silk ligature was cut and removed from the heart. The chest was closed while the heart was reperfused. Two weeks after the LAD ligature surgery, LV functions were further evaluated using echocardiography.

Echocardiography for left ventricular (LV) functions

LV functions were measured using echocardiography after LAD ligature surgery for 2 weeks. During the measurement, animals were maintained in a core temperature of 37°C, and anesthetized by inhalation of 1.2%-1.5% isoflurane in oxygen. Transthoracic M-mode images of the LV in parasternal short-axis views were obtained at the position of the papillary muscles using a high-resolution ultrasound probe. The parameters include ejection fraction (EF), fractional shortening (FS), LV internal dimension in diastole (LVIDd) and LV internal dimension in systole (LVIDs) were measured.

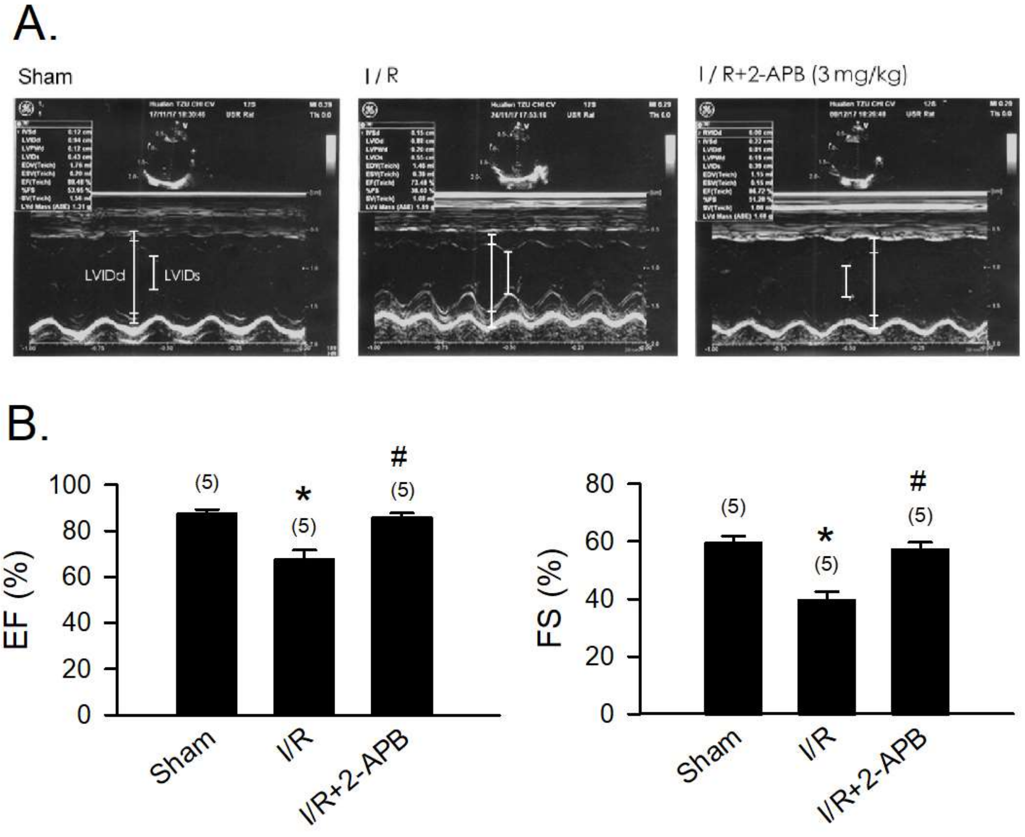

Figure S1. 2-APB improves the recovery of contractile functions in rat hearts after I/R injury. (A) Representative images of cardiac function determined by echocardiography. (B) I/R reduced the EF and FS, and these effects were abolished by 2-APB. EF (%), and FS (%) were determined by echocardiography. Values shown in parenthesis represent the number in

each group. *$p < 0.05$ different from the sham group; #$p < 0.05$ different from the I/R group. Data are expressed as mean ± SEM. EF, ejection fraction; FS, fractional shortening; I/R, ischemia/reperfusion.
